# Mechanisms of systemic immune modulation in tumor development: Interactions across lymph nodes, bone marrow, and blood

**DOI:** 10.1007/s10555-026-10383-6

**Published:** 2026-10-01

**Authors:** Xiaofei Yu, Linh Phạm, Tanja D. de Gruijl, Marieke F. Fransen, Febe van Maldegem

**Affiliations:** 1 Molecular Cell Biology and Immunology, Amsterdam UMC, Location Vrije Universiteit Amsterdam, Amsterdam, The Netherlands; 2 Amsterdam Institute for Immunology and Infectious Diseases, Amsterdam, The Netherlands; 3 Cancer Center Amsterdam, Amsterdam, The Netherlands; 4 Medical Oncology, Amsterdam UMC, Location Vrije Universiteit Amsterdam, Amsterdam, The Netherlands; 5 Pulmonary Medicine, Amsterdam UMC, Location Vrije Universiteit Amsterdam, Amsterdam, The Netherlands

**Keywords:** Tumor-draining lymph nodes, Bone marrow, Peripheral blood, Systemic immune remodeling, Cancer metastasis, Immunotherapy response

## Abstract

Cancer is a systemic disease in which the tumor interacts with the entirety of the body to promote tumor pathogenesis and progression. While tremendous efforts have focused on understanding the local immune responses in the primary site because of the noticeable immunosuppressive characteristics in the tumor microenvironment (TME), the interaction between the tumor and the host’s systemic response has not received much attention. These interactions influence primary tumor growth and treatment responses and can further facilitate tumor dissemination and condition distant tissues for metastatic colonization. In this review, we summarize the reciprocal interactions between the primary tumor and the systemic environment throughout cancer development, including pre-metastatic niche formation and metastatic progression, with particular emphasis on tumor-draining lymph nodes (TDLNs), bone marrow (BM), and blood circulation. By integrating these perspectives, this review aims to provide a more comprehensive understanding of tumor development within the whole-body context, thereby highlighting the development and optimization of strategies in cancer diagnosis, prognosis, and treatment that account for the systemic nature of the disease.

## Introduction

Over the last decades, particularly since the arrival of immune checkpoint inhibitors, it has become overwhelmingly clear that tumors actively reshape the surrounding tumor microenvironment (TME) by recruiting and reprogramming myeloid cells, fibroblasts, and other stromal components, thereby establishing an immunosuppressive environment that supports tumor growth and progression [1]. Importantly, this remodeling can extend beyond the primary tumor site. In 2010, McAllister and Weinberg proposed the concept of “tumor instigation,” which suggests that tumors actively disrupt the host environment by secreting factors with systemic effects, thereby promoting tumor growth and distant metastasis [2]. This recognition has contributed to a broader view of cancer as a disease with systemic consequences, rather than a purely localized process. Tumors and their surrounding microenvironment continuously release cytokines, chemokines, growth factors, extracellular vesicles, and other signals that can influence distant immune organs and tissues through lymphatic and vascular routes. Thus, the primary tumor is not only a major site of local tumor-immune interaction, but also the initiation point of systemic immune regulation.

Studies have further demonstrated that this systemic regulation involves multiple tissues and compartments within the systemic circulation. For example, the activation and initiation of T cell responses predominantly take place within lymphoid organs while tumor-associated myeloid cells are recruited from bone marrow progenitors [3, 4]. Conditioning of the pre-metastatic niche (PMN) is another example of the ability of the tumor to perturb distant organs [5]. Primary tumors release soluble mediators and extracellular vesicles that remodel hematopoiesis, mobilize immune cells, and alter local environments in distant tissues, collectively establishing pre-metastatic niches that facilitate the subsequent survival and colonization of disseminated tumor cells [5–8]. Tumor-draining lymph nodes (TDLNs) can similarly undergo lymphangiogenic, stromal, and immune remodeling and, in turn, influence immune responses within the primary tumor [9, 10]. The circulation provides an additional environment in which tumor cells interact with platelets and immune cells to enhance their survival and metastatic competence [11]. Thus, TDLNs, bone marrow, blood, and distant tissues constitute an interconnected systemic network through which primary tumors can influence local progression, invasion, and metastatic spread.

**Fig. 1:**
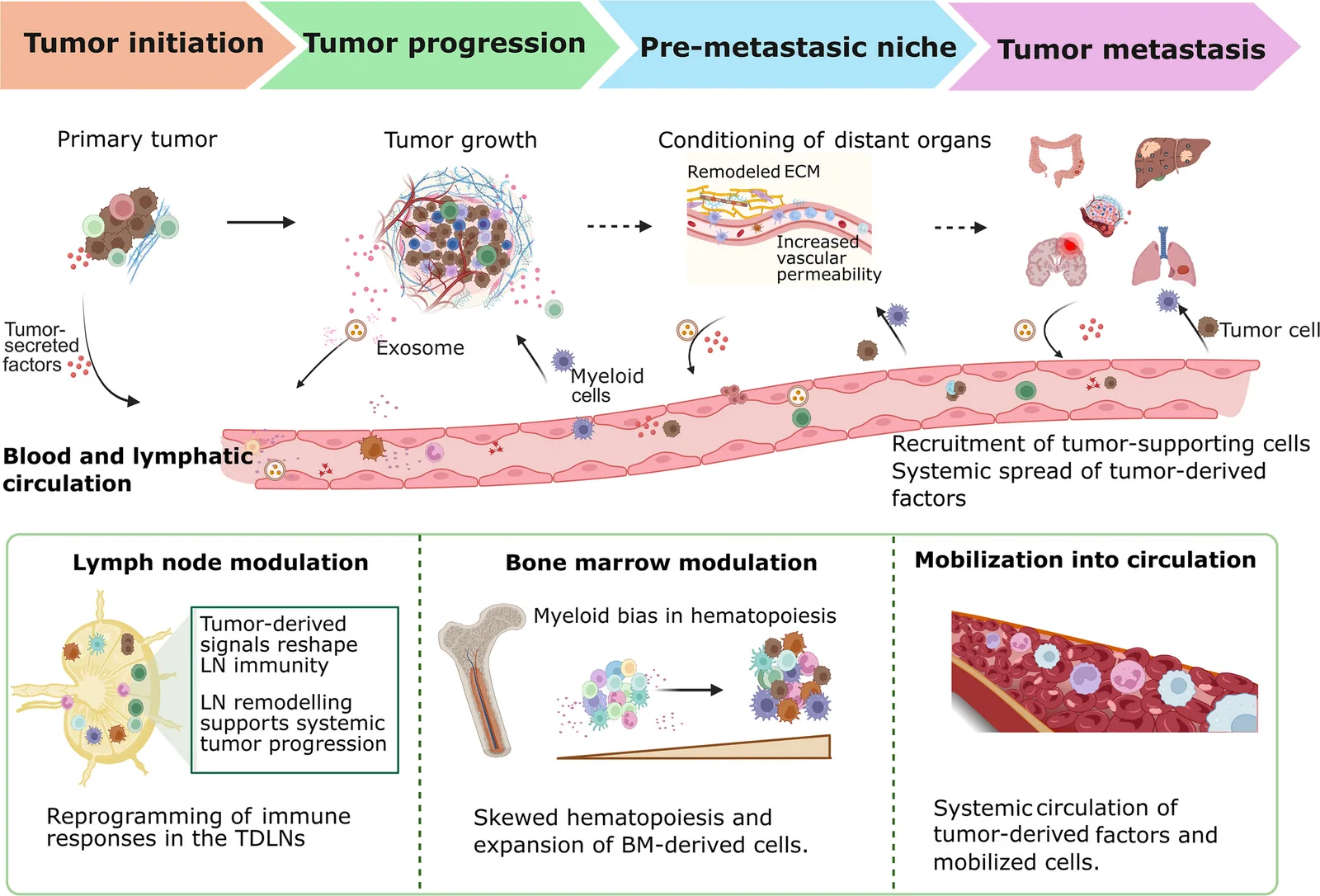
Reciprocal interactions among tumor-draining lymph nodes, bone marrow, and the circulation during tumor development. Tumor-derived signals remodel tumor-draining lymph nodes (TDLNs) and bone marrow hematopoiesis, thereby altering the composition and functional state of circulating immune cells. In turn, blood-borne cells and mediators can feed back to further reshape immune responses within TDLNs and the bone marrow. Together, these reciprocal interactions contribute to tumor growth, pre-metastatic niche formation, and metastatic progression. Created with BioRender.com

Our review focuses on the interactions between primary tumors and the host systemic immune environment during tumor development (Fig. 1), aiming to provide a cross-tissue, systems-level perspective and to highlight considerations for immunotherapeutic strategies targeting the systemic features of the disease.

## Tumor-draining lymph nodes as sentinel and stepping stone

**Fig. 2:**
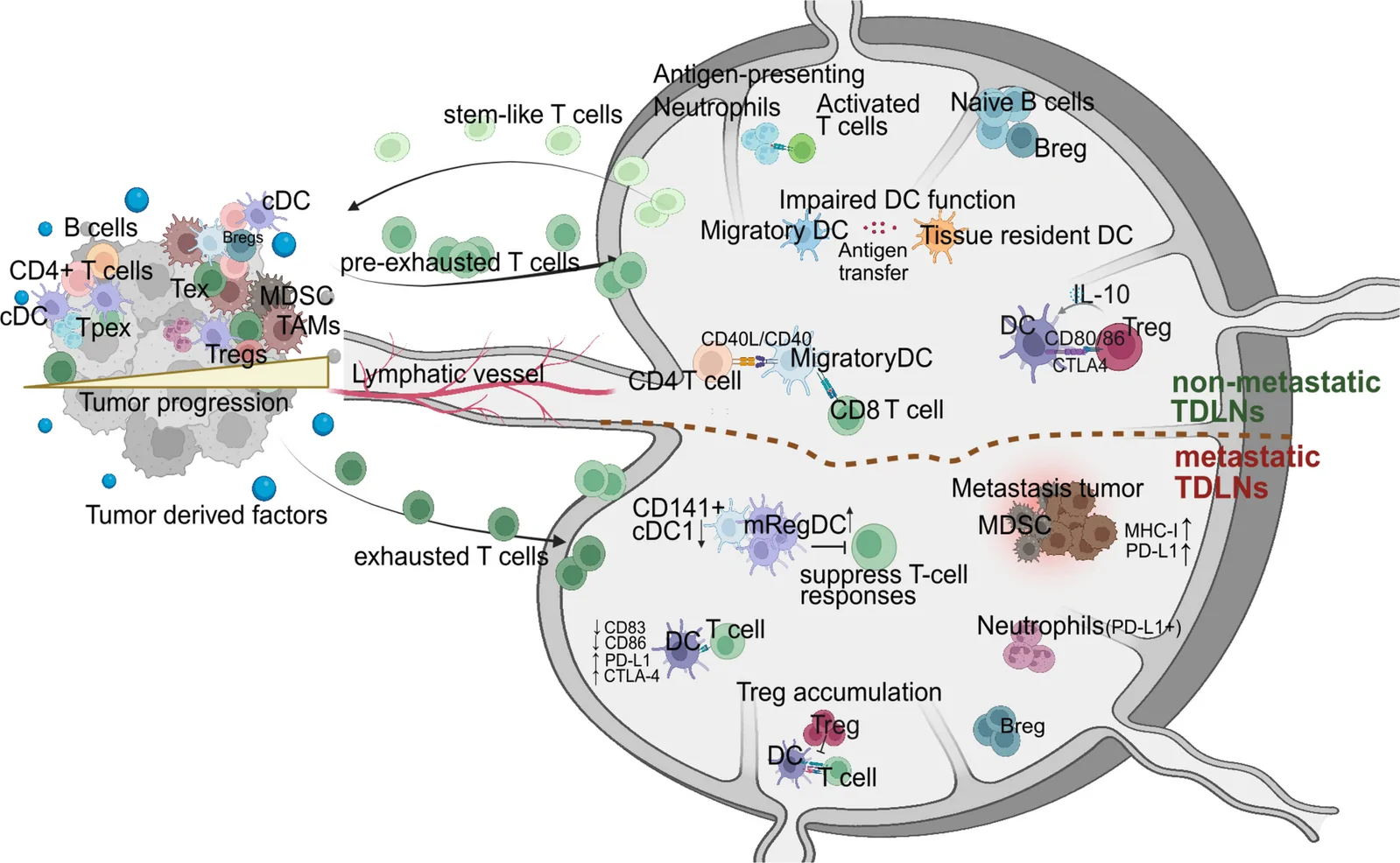
Dynamic immune remodeling in tumor and tumor-draining lymph nodes (TDLNs) during tumor progression. TDLNs serve as a long-term reservoir of stem-like exhausted T cells and continuously generate progenitor-exhausted CD8⁺ T cells (Tpex). These Tpex migrate into tumors and gradually differentiate into terminally exhausted cells (Tex) under chronic antigen stimulation. In non-metastatic TDLNs, migratory cDCs (CD11c^low^ MHCII^+^) interact with CD4^+^ T cells through CD40-CD40L contact, upregulate the co-stimulatory signal and initiate a tumor-specific CD8^+^ T cell response. Tumor antigens acquired by migratory DCs can be transferred to tissue-resident dendritic cells, enabling efficient antigen cross-presentation and sustained CD8⁺ T cell priming, although this process may be impaired by tumor-induced immune suppression. Once the cancer cells can be detected in TDLNs, the immune microenvironment gradually shifts toward an immunosuppressive state. Regulatory DC populations, together with regulatory T cells and regulatory B cells, accumulate within the lymph node and collectively dampen DC activation and T cell effector functions. Created with BioRender.com

Lymph nodes form a critical immune surveillance network, maintaining immune homeostasis by filtering antigens from lymph fluid and activating T and B lymphocytes [12]. Antigens may either be transported to the lymph node by migratory dendritic cells or drain directly through the lymph and subsequently be captured and presented by LN-resident antigen-presenting cells (LN-resident APCs) [13]. TDLNs directly receive lymphatic efflux from the tumor bed and constitute key sentinel sites for tumor-associated immune responses [14]. Exposure to tumor antigens and various immunosuppressive factors secreted by the tumor will reshape the immune microenvironment of the TDLNs [15]. Within non-metastatic TDLNs, early innate immune sensing and antigen presentation can initiate antitumor adaptive immune responses, while immunoregulatory and suppressive programs may also begin to emerge at this stage [16], potentially initiated or amplified by tumor-conditioned DCs and other APCs [17, 18]. As the primary tumor progresses, the balance between immune activation and immune suppression gradually shifts toward immune dysfunction and immunosuppression. Once metastatic tumor cells reach the draining lymph node, additional lymphatic, stromal, and immune remodeling further reshapes the TDLN microenvironment and promotes a more immunosuppressive and metastasis-supportive state (Fig. 2). Thus, TDLNs undergo dynamic alterations in both cellular composition and functional states throughout tumor development, which can in turn affect the primary tumor site, promoting tumor progression and influencing responses to immunotherapy [14, 19]. These dynamic changes in TDLNs should therefore be considered when selecting therapeutic strategies and determining the optimal timing of intervention.

The earliest immune changes within TDLNs remain difficult to define. Tumor initiation and early progression form a continuous process without a clear biological boundary, and this is particularly difficult to study in patients because most surgically resected tumors are obtained after substantial disease progression. Mouse studies provide valuable insights: cell line transplant models show that lymph nodes can sense and respond to tumor-associated antigens within days of tumor onset [20, 21]. However, our understanding of very early TDLN-initiated immune responses remains limited, largely because of the constraints of current experimental models. In transplant models, the death of many inoculated cells releases DAMPs that activate dendritic cells, artificially amplifying the immune response [22]. In spontaneous models, precise control of tumor initiation often requires viral vectors, which themselves can trigger immune responses and confound observations of physiological early antitumor immunity [23, 24]. Additionally, collecting lymph nodes at very early tumor stages in mice is practically challenging. We therefore discuss tumor initiation and early progression together within the non-metastatic TDLN section, followed by a separate discussion of the further immune alterations that accompany lymph node metastasis.

### Early immune sensing and remodeling in non-metastatic TDLNs

TDLNs contain diverse innate and adaptive immune populations that are progressively reshaped during tumor development. Innate immune cells, including macrophages, neutrophils, and dendritic cells, contribute to early tumor sensing and antigen presentation, followed by adaptive T- and B-cell responses and the emergence of immunoregulatory mechanisms.

In non-metastatic TDLNs, CD169⁺ macrophages reside predominantly in the subcapsular sinus, where their strategic position enables them to serve as an early interface for lymph-borne tumor-derived material entering through afferent lymphatic vessels [25]. In melanoma models, tumor-derived extracellular vesicles (TEVs) transported through lymphatics are initially captured by these CD169⁺ subcapsular sinus macrophages in TDLNs, which restrict their further dissemination into the lymph node cortex. Consistent with a protective role of sinus macrophages, a higher density of CD169⁺ macrophages in regional lymph nodes of breast cancer patients has been associated with earlier clinical stage and absence of lymph node metastasis [26]. However, as tumors progress, disruption of this macrophage barrier permits increased vesicle-B cell interactions, thereby promoting tumor-supportive humoral immunity [25].

Neutrophils are known for their rapid response to infection. Upon inflammatory events, lymph-derived neutrophils can reach lymph nodes within minutes, primarily accumulating in the subcapsular sinuses; hematogenous neutrophils arrive 3 days later [27]. A similar early neutrophil migration is likely to occur during the initial stages of tumorigenesis. In non-metastatic TDLNs (N0), neutrophils displayed an antigen-presenting phenotype (HLA-DR⁺, CD80⁺, CD86⁺, ICAM-1⁺, PD-L1⁻) and were associated with activation of T cells, suggestive of a role in initiating anti-tumor adaptive immunity [28]. Together, these early innate responses provide the first layer of tumor sensing within TDLNs and create the conditions for subsequent antigen presentation and adaptive immune activation.

Activated antigen-presenting cells express high levels of C–C chemokine receptor type 7 (CCR7), which enables their migration through lymphatic vessels into the TDLNs, where conventional DCs (cDCs) predominate [14]. Tumor-derived antigens are primarily presented by these cDCs to CD8⁺ T cells, and when also offering co-stimulatory signals mediated by CD80 and CD86, the cDCs can drive the full activation and functional differentiation of the tumor-specific CD8 T cells [29]. cDCs can be either migratory or LN-resident. Migratory cDCs transport tumor antigens to draining lymph nodes and can directly prime tumor-specific CD8 T cells, while also transferring antigen to LN-resident cDCs that further amplify and sustain T-cell activation [30]. Migratory cDCs and LN-resident DCs include cDC1- and cDC2-like subsets, with cDC1 generally specialized in cross-presenting antigen and priming CD8 T cells and cDC2 more commonly in priming and differentiating CD4^+^ T cells [31]. A recent study indicates that a specific CD11c^low^MHC-II^hi^ migratory DC population drives tumor-specific CD4 T cell responses. They can be distinguished from other DCs in the TDLNs by their expression of maturation markers such as CD80, CD86, and CD40, as well as genes involved in migration, adhesion, and T cell chemotaxis [32]. These migratory DCs arrive in the lymph node in a poorly activated transitional state between cDC2 and mature DC phenotypes, enriched in immunoregulatory molecules (mRegDC) . After interacting with CD4^ +^ T cells, particularly through CD40-CD40L contact in the tumor-draining lymph node, these dendritic cells undergo functional licensing, upregulate costimulatory molecules, and become capable of initiating tumor-specific T cell responses [32]. However, with tumor growth, the impairment of DC function in TDLNs can arise even before lymph node metastasis occurs, which is evident from downregulation of CD80/CD86 and reduced IL-12 production [17, 18] and precedes functional impairment of T-cell activation and Treg expansion [22]. In melanoma, the associated clinical outcomes appear to be DC subset-specific: reduced frequencies of migratory DCs were primarily associated with local recurrence, whereas impaired maturation of lymph node-resident DCs was linked to subsequent distant recurrence and melanoma-specific survival [18].

TDLNs are considered as the primary reservoir for tumor-specific CD8⁺ T cells, including a TCF-1⁺ stem-like/progenitor population with self-renewal capacity and the ability to generate differentiated effector T cells, thereby supporting sustained antitumor immunity and responses to immune checkpoint blockade [10, 14, 33]. In a spontaneous lung adenocarcinoma model, Schenkel et al. tracked the dynamics of tumor-specific TCF-1⁺ CD8⁺ T cells between the tumor and TDLNs and found that the same clones, or those with similar proportions, remained relatively stable. Furthermore, blocking lymphocyte egress from the TDLNs reduced the number of TCF-1⁺ CD8⁺ T cells within the tumor, indicating that TDLNs serve as a major source of these TCF-1⁺ CD8⁺ T cells and that their loss compromises antitumor immunity [10, 34]. Consistent with these preclinical findings, TCR analyses from several human scRNA-seq studies encompassing tumors and lymph nodes have demonstrated that TDLN-derived T cell clones can evolve into effector or exhausted lineages within the tumor, collectively supporting that T cells are activated in the TDLNs, migrate into the tumor, and continuously circulate between the two compartments [35, 36]. Importantly, PD-1 blockade primarily activates newly recruited T cell clones originating from peripheral or lymph node compartments, leading to expansion of progenitor-exhausted CD8⁺ T cells (Tpex), characterized by low expression of coinhibitory molecules and high expression of GZMK, rather than reactivating pre-existing exhausted T cells within the tumor [37].

TDLN B cell follicles, in a melanoma study, were found largely preserved, including naive B cells, germinal center B cells, and plasma cell precursors, resembling the architecture of immunologically active but undisturbed secondary lymphoid organs [38]. However, another study on oral squamous cell carcinoma found that non-metastatic TDLNs contained a higher proportion of naive B cells alongside an increased frequency of IL-10 producing regulatory B cells [39]. Clonal tracing confirmed a clonal relationship between class switched memory B cells in the lymph nodes and memory B cells and plasma cells in the tumor of high-grade serous ovarian cancer [40]. Furthermore, there is a growing body of evidence suggesting that the immunological state of B cells and CD4⁺ T helper cells in TDLNs is linked to the development or maturation of tertiary lymphoid structures (TLS) within tumors. TLS are generally linked to favorable clinical outcomes and improved responses to antitumor immunotherapies. In NSCLC it was shown that TDLN metastasis resulted in fewer B cells in the primary tumor, less germinal center formation in the TLS and reduced predictive value for the presence of TLS in the tumor [41]. However, these antitumor responses do not remain unopposed. As the tumor progresses, immunoregulatory mechanisms increasingly emerge within TDLNs and constrain effective T-cell activation.

Regulatory programs can emerge within TDLNs at relatively early stages of tumor development. Genetically engineered lung adenocarcinoma models suggest that T-cell anergy and peripheral regulatory T cell (Treg) differentiation can also be initiated at very early stages [16]. Consistent with this, Tregs expand within TDLNs in multiple preclinical models and human tumors [38, 42, 43]. This expansion may result from the trafficking of tumor-associated Tregs between the tumor microenvironment and draining lymph nodes as well as local proliferation [44–47]. Tregs can suppress DC maturation and antigen presentation through mechanisms such as IL-10 secretion and CTLA-4 mediated trans-endocytosis of CD80 and CD86, thereby impairing TH1 differentiation and CD8⁺ T cell activation [48]. Conversely, tumor-conditioned DCs may promote the induction or expansion of Tregs in TDLNs, establishing a reciprocal immunosuppressive circuit. Recent studies have also highlighted the impact of cellular spatial organization on the balance between effector and suppressive immunity within lymph nodes. In non-metastatic lymph nodes, Tpex and intermediate-exhausted CD8^+^ T cells (Tex-int) are closely colocalized with CD4⁺ T cells, Tregs, and DCs, consistent with local activation and differentiation of CD8⁺ T cells [49].

In summary, non-metastatic TDLNs initially support tumor sensing, antigen presentation, and adaptive immune activation, while immunoregulatory programs progressively emerge as tumor-derived signals persist. This gradual shift from immune activation toward immune dysfunction provides the basis for the more profound immunosuppressive and structural remodeling that accompanies lymph node metastasis, as discussed in the following section.

### Immune suppression and structural remodeling in metastatic TDLNs

Lymph node metastasis is a sign of tumor progression; however, in clinical practice, metastasis into lymph nodes does not always indicate advanced-stage disease. For many types of tumors, LN metastases can appear already in early clinical stages, suggesting that lymph nodes play an important role in the early spread of tumor cells. Distant metastases can indeed arise via LN metastases, as determined by shared clonality, but can also occur independently of lymph node involvement, through blood dissemination [50, 51]. Even when distant lesions are not directly seeded by LN-resident tumor cells, LN colonization may facilitate their establishment by inducing systemic tumor-specific immune tolerance [52]. LN metastasis can have a significant impact on the prognosis of the disease, as it reflects the fact that the tumor cells have begun to break through the confines of the primary site, thereby increasing the risk of distant organ metastasis and influencing survival outcomes. Reticker-Flynn et al. proposed that primary tumors simultaneously upregulate MHC-I and PD-L1 molecules in response to chronic IFN signaling, enhancing initial dissemination to LNs by promoting NK cell evasion and inhibiting T cell responses, respectively [52]. Colonization of TDLNs by cancer cells drives a remodeling of the antigen-presenting network: the antigen-presenting function of DCs becomes further impaired, characterized by downregulation of the costimulatory molecules CD83 and CD86, and upregulation of the inhibitory molecules PD-L1 (Fig. 2) [17, 18, 49, 53]. Meanwhile, mature and cross-presenting DCs (CD141⁺ cDC1) markedly decrease, whereas LAMP3⁺CCR7⁺PD-L1⁺ mregDCs emerge as the dominant myeloid population. These DCs suppress T-cell responses and facilitate metastatic progression [38]. In addition to these changes, the accumulation of monocytic myeloid-derived suppressor cells (M-MDSCs), immunosuppressive PD-L1 + neutrophils, and increased cytokine and chemokine secretion are also important features of immune suppression in TDLNs affected by metastasis [17, 28, 53, 54].

Consistent with the alterations in APC maturation, metastatic TDLNs contain markedly fewer Tpex cells and more terminally exhausted CD8⁺ T cells, accompanied by reduced effector function and poor responsiveness to immunotherapy [49, 55]. The remaining Tpex and Tex-int cells are located within immunosuppressive niches enriched in proliferating Tregs, naïve or quiescent CD4⁺ T cells, and DCs expressing immunoregulatory molecules [49]. In parallel, Treg and IL-10-producing Breg populations increase in frequency and exhibit stronger immunosuppressive transcriptional programs, collectively reinforcing the suppressive immune landscape within metastatic TDLNs [39, 44].

Importantly, the changes induced by metastasis into the lymph node are predictive of outcome, as an immune suppressive gene signature in the non-malignant areas of the TDLNs was correlated with disease recurrence in melanoma after surgical resection of the primary tumor and TDLNs [56]. Beyond immune modulation, invasive tumor cells profoundly reshape the TDLN microenvironment through structural remodeling. This includes proliferation and functional reprogramming of fibroblastic reticular cells (FRCs) [57], as well as extensive remodeling of the lymph-node vasculature [58]. Such remodeling enhances the capacity of tumor cells to enter the lymphatic circulation and facilitates their dissemination to distant tissues via lymphatic routes [59, 60].

Tumor cells within TDLNs are required to adapt and acquire immune evasion mechanisms, giving rise to more aggressive and immune-resistant phenotypes. Using a mouse mammary tumor model, Shih-Chia Huang et al. found that TDLN-derived mammary tumor cells have enhanced colony-forming capacity and accelerated tumor growth upon re-implantation [61]. Consistent with these preclinical observations, gene expression profiling showed that human metastatic breast and bladder tumor cells within lymph nodes possess enhanced immune evasion capability [62]. Collectively, TDLNs are not only the source of antitumor immune responses and a site of tumor cell dissemination but also dynamic niches that are modulated to drive the immune escape and malignant evolution of cancer cells, ultimately facilitating distant metastasis and shaping disease progression.

### Targeting TDLN immunity to enhance systemic antitumor responses

The clinical management of TDLNs requires a careful balance between oncological control and preservation of antitumor immunity. Clinical evidence suggests that extensive lymphadenectomy may compromise subsequent responses to immune checkpoint blockade, supporting a more selective approach that avoids unnecessary removal of immunologically functional lymph nodes [63, 64]. Nevertheless, clinically involved or metastatic TDLNs may still require local treatment to achieve adequate regional disease control and the optimal duration of TDLN preservation remains undefined. Preclinical evidence suggests that maintaining functional TDLNs during the initial phase of immune priming, followed by delayed nodal treatment, may preserve systemic antitumor immunity without permanently foregoing regional tumor control [65]. This raises the possibility of a preoperative therapeutic window in which immunostimulatory therapies could be administered while the tumor-TDLNs axis remains intact. Neoadjuvant immune checkpoint blockade has demonstrated improved clinical outcomes across multiple solid tumors, including melanoma and NSCLC, supporting the therapeutic potential of initiating immunotherapy before surgical removal of the tumor and draining lymph nodes [66, 67].

Given the important role of TDLNs in shaping antitumor immunity, increasing efforts have focused on therapeutically modulating immune responses within TDLNs to enhance systemic tumor control with fewer side effects. One major strategy involves enhancing DC function within TDLNs. DC targeted agents, such as Flt3L combined with CD40 stimulation, TLR9 agonists, or GM-CSF, aim to enhance DC activation and antigen-presenting capacity, thereby improving tumor control and increasing the abundance of TCF-1⁺ CD8⁺ T cells [34, 68–70]. Importantly, in melanoma, therapeutic efficacy was evident after the LN was removed one week after TLR9 agonist injection, suggesting that only a brief window is needed to reinvigorate suboptimally primed T cells in TDLNs [70]. In parallel, immune checkpoint blockade relieves inhibitory signaling, thereby reshaping T-cell differentiation within TDLNs. Neoadjuvant PD-L1 blockade promotes the differentiation of progenitor-exhausted CD8⁺ T cells (Tpex) into intermediate-exhausted CD8⁺ T cells (Tex-int) [49]. It also remodels the spatial architecture of TDLNs, increasing the density of DCs surrounding both Tpex and Tex-int populations [49]. Subsequently, Tex-int cells enter the circulation and migrate into tumors, where they exert cytotoxic functions. In metastatic LNs, Tpex and Tex-int are instead embedded in tolerogenic DC and suppressive Treg-rich niches [49]. CTLA-4 blockade enhances the interaction between CD4⁺ T cells and cDC2s in TDLNs, activating the CD40-CD40L signaling axis [32]. This interaction upregulates costimulatory molecules (CD80, CD86) and immunoregulatory mediators such as IL-27 in cDC2s, ultimately amplifying antitumor immune responses [32]. Local delivery of anti-CTLA-4 antibodies to lymphatic drainage areas following surgical resection of primary melanoma has been shown to activate migratory DC subsets within sentinel lymph nodes, reduce Treg frequencies, and induce the activation of effector T cells in the peripheral blood [71].

It is also important to consider how chemotherapy and radiotherapy affect lymph node immunity. In preclinical models, adjuvant chemotherapy can suppress radiotherapy-induced T-cell responses [72]. Radiotherapy can also directly affect TDLN immunity: irradiation of TDLNs impairs T-cell priming and systemic antitumor immunity in preclinical models [73], while clinical studies suggest that these effects may depend on radiation dose and nodal exposure [74]. Irradiation of non-involved TDLNs has also been associated with poorer progression-free survival in NSCLC [75]. However, the contribution of distant, non-tumor-draining LNs to systemic antitumor immunity remains less well understood. Under intact lymphatic drainage, immune activation following PD-1/PD-L1 blockade occurs predominantly within TDLNs rather than non-draining LNs [76]. Currently, there is little evidence that distant LNs compensate for TDLN removal; they may become immunologically relevant if they subsequently drain emerging metastatic lesions, although immune suppression may be more profound in this setting. It will be critical to understand how other therapies that may be given in combination with immune checkpoint inhibitors may impact (possibly conserve) lymph node function, such as chemo and radiotherapy.

## Tumor-driven myeloid mobilization and recruitment from the bone marrow

### Mobilization and recruitment of bone marrow-derived cells

The bone marrow is the primary site of hematopoiesis and harbors a complex and highly organized microenvironment that supports the self-renewal and differentiation of hematopoietic stem cells (HSCs) to maintain a constant supply of blood cells [77]. HSCs give rise to common lymphoid progenitors (CLPs) and common myeloid progenitors (CMPs). CLPs further differentiate into T, B and NK cells, whereas CMPs give rise to granulocyte-monocyte progenitors (GMPs), monocyte-dendritic progenitors (MDPs), as well as megakaryocyte-erythroid progenitors. GMPs can further commit to the granulocytic or monocytic lineages, a process driven by hematopoietic growth factors such as granulocyte colony-stimulating factor (G-CSF), granulocyte–macrophage colony-stimulating factor (GM-CSF) and macrophage colony-stimulating factor (M-CSF) [78]. MDPs possess dual differentiation potential and can give rise either to monocytes or to common dendritic progenitors (CDPs), which subsequently develop into plasmacytoid dendritic cells (pDCs) and conventional dendritic cells (cDCs) [79].

**Fig. 3:**
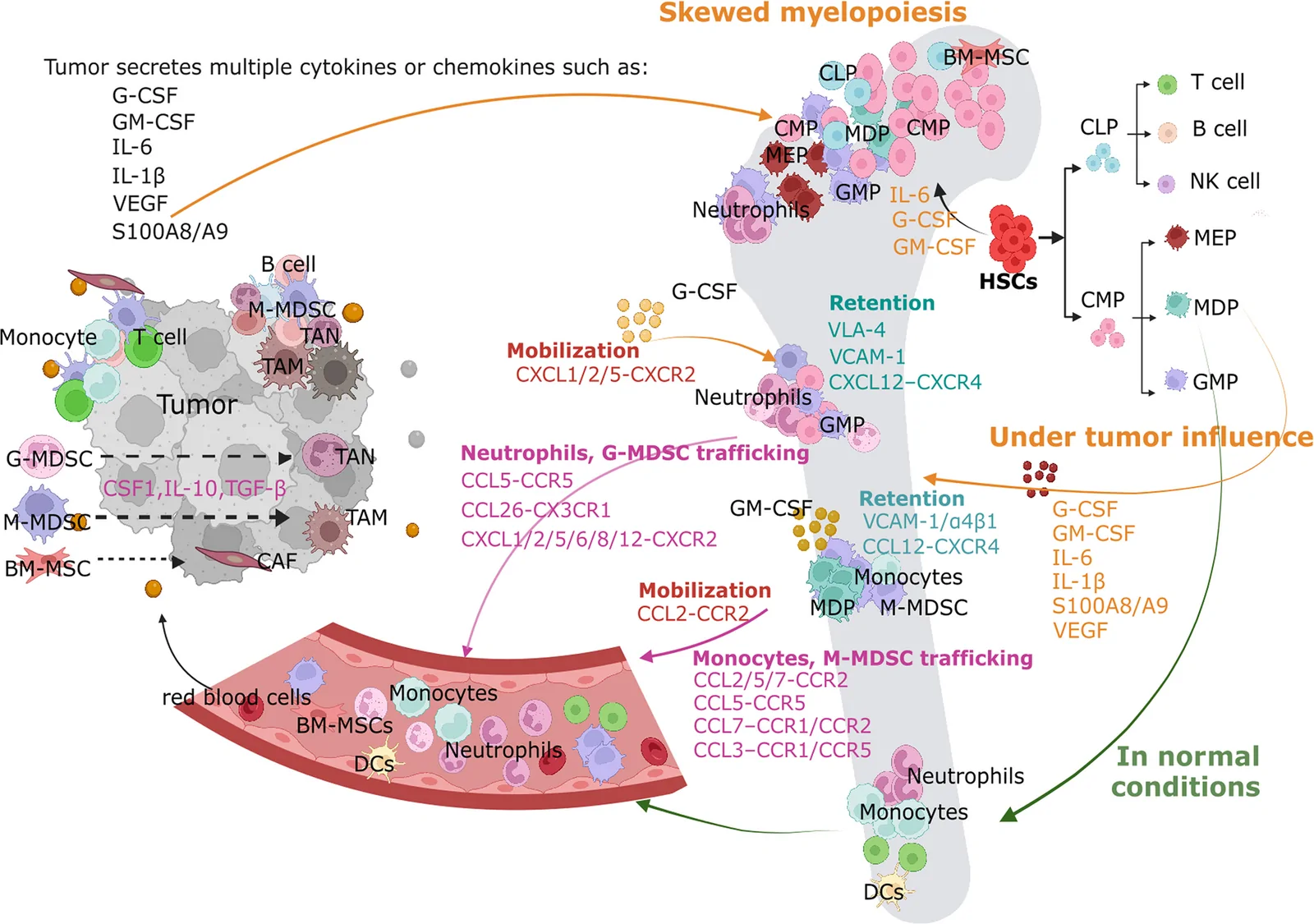
Tumor-induced hematopoietic reprogramming in bone marrow and systemic myeloid mobilization. Tumor-derived cytokines such as IL-6, G-CSF, and GM-CSF act on hematopoietic stem and progenitor cells (HSPCs), skewing differentiation toward myeloid-biased lineages and expanding common myeloid progenitors (CMPs). Under normal conditions, CMPs differentiate into mature neutrophils, monocytes and DCs; however, in the presence of tumors, tumor-associated factors, including G-CSF/GM-CSF/IL-6/IL-1β, disrupt bone marrow retention by downregulating CXCR4 and the CXCL12-CXCR4/VCAM-1/VLA-4 axis, thereby promoting CXCR2-dependent granulocytic mobilization through CXCL1/2/5-CXCR2 signaling. In parallel, GM-CSF and IL-6 facilitate the generation and premature egress of immature myeloid-derived suppressor cells (MDSCs) from the bone marrow. Within the circulation, neutrophils and granulocytic MDSCs (G-MDSCs) are guided by CXCR2 ligands (CXCL1/2/5/8), whereas monocytic MDSCs (M-MDSCs) migrate via CCR2-, CCR1-, and CCR5-mediated pathways in response to CCL2, CCL7, CCL5, and CCL3. In the tumor microenvironment, these cells differentiate into immunosuppressive tumor-associated macrophages (TAMs) and tumor-associated neutrophils (TANs) under the influence of CSF1, IL-10, and TGF-β, contributing to immune evasion and tumor progression. Created with BioRender

Under homeostatic conditions, mature neutrophils and monocytes are mobilized directly from the bone marrow into the peripheral circulation. Monocytes subsequently complete their differentiation within peripheral tissues. During tumor development, diverse soluble factors produced by the tumor and its stromal components can influence multiple processes, including hematopoiesis, mobilization into the bloodstream, homing to the tumor site, and retention within the tumor microenvironment, thereby exerting profound effects on the circulation of myeloid cells (Fig. 3) [80]. Consequently, in cancer patients, mature neutrophils, mature monocytes, and immature myeloid-derived suppressor cells (MDSCs) coexist systemically, with MDSC levels progressively increasing as the disease advances [81]. Meanwhile, tumor-induced hematopoietic skewing also suppresses lymphoid differentiation [82, 83]. In addition, tumor-associated inflammatory factors can directly interfere with DC lineage commitment. For instance, tumor-derived IL-6 acts on CDPs in the bone marrow, inducing transcription factor C/EBPβ and sustaining Zeb2 expression, which markedly reduces cDC1 generation [84]. In line with this, analyses of human pancreatic ductal adenocarcinoma (PDAC) have shown a significant decrease in both peripheral and bone marrow cDC2 populations and their progenitors, with the reduction of bone marrow cDC2 correlating negatively with elevated serum IL-6 levels [85].

Mature neutrophils are among the most abundant circulating myeloid cells in steady state [86]. Cancer patients harbor large numbers of immature granulocytic myeloid-derived suppressor cells (G-MDSCs), both in circulation and in the tumor microenvironment (where they may comprise tumor-associated neutrophils (TANs)) [87, 88]. Recent work has further demonstrated that tumor-induced granulocytic reprogramming begins at an early stage of hematopoiesis. In breast cancer models, tumors accelerated the commitment of HSPCs toward myeloid and neutrophil differentiation at the expense of lymphoid and erythroid development, without generating a distinct myeloid lineage [89]. IL-1β was identified as an important mediator of this process, and its blockade normalized tumor-altered granulopoiesis, reduced the immunosuppressive phenotype of neutrophils, and limited metastatic spread [89]. Importantly, tumor-induced hematopoietic alterations may persist even after removal of the primary tumor. In breast and lung tumor models, TEVs promoted MMP-13 expression in bone marrow osteoprogenitors, leading to persistent CD41⁻ GMP expansion, excessive myelopoiesis and systemic immunosuppression after tumor resection [90]. Pharmacological inhibition of MMP-13 following tumor resection accelerated restoration of hematopoietic homeostasis and improved subsequent responses to immunotherapy [90].

Beyond reprogramming of granulopoiesis at the progenitor level, tumors also regulate the subsequent retention and mobilization of granulocytic cells from the bone marrow. Within the bone marrow, tumor-derived hematopoietic factors, particularly G-CSF, disrupt the balance between retention and mobilization of granulocytic cells. Under physiological conditions, the CXCL12-CXCR4 axis and adhesion molecules such as VCAM-1 and VLA-4 maintain neutrophil retention within the bone marrow. Tumor-derived G-CSF downregulates CXCR4 expression on granulocytes while tumor cells themselves or tumor-associated fibroblasts can secrete CXCR2 ligands (CXCL1, CXCL2, CXCL5), creating a “mobilization pressure” that promotes premature egress of granulocytes into circulation [91]. Circulating human neutrophils and G-MDSCs are mainly attracted to tumor sites by the CXCR2 ligands CXCL1, CXCL2, CXCL5, CXCL6, and CXCL8/IL-8 [87]. Other chemokine axes, including CCL5-CCR5 and CCL26-CX3CR1, may also contribute, though their roles appear less prominent than that of CXCR2-mediated signaling [88]. In KRAS-driven tumor models, tumor cells further enhance GM-CSF and CXCL1/2/5 secretion, amplifying CXCR2-dependent granulocytic release and leading to systemic accumulation of immature granulocytes [92, 93].

Tumor-associated macrophages (TAMs) constitute the prime cell population that establishes the immunosuppressive tumor microenvironment. Due to the limited number of tissue-resident macrophages, sustained tumor growth relies heavily on the continuous recruitment of peripheral monocytes. This enhanced and prolonged recruitment pressure exerts a reverse effect on the hematopoietic system in the bone marrow. The precursors of monocytes primarily reside within the bone marrow cavity, where they are anchored to osteoblasts through VCAM-1/α4β1 integrin-mediated cell matrix interactions, maintaining a quiescent state. Tumor-derived PTHrP activates PTH1R in osteoblasts, inducing IL-6- and VEGF-A-dependent Src family kinase signaling in monocytic myeloid-derived suppressor cells (M-MDSCs). This disrupts VLA-4-VCAM1-mediated retention and promotes M-MDSC mobilization from the bone marrow into the circulation [94]. During peripheral migration, the CCL2-CCR2 axis serves as the core pathway driving the mobilization and tumor homing of monocytes and M-MDSCs. Tumor-derived chemokines such as CCL2, CCL7, and CCL5 bind to CCR2 to promote monocyte and M-MDSC egress from the bone marrow and targeted recruitment to the tumor (Fig. 3) [95]. CCR2 blockade significantly reduces monocyte infiltration, delays metastatic outgrowth, and enhances antitumor immune responses [96]. Notably, in steady-state and certain chronic inflammatory settings, chemokine receptors such as CCR1, CCR2, and CCR5 exhibit a degree of complementarity in mediating the tissue infiltration of monocytic lineage cells [97]. In gliomas, CCL2 and CCL7 exhibit functional redundancy in driving the migration of CCR2⁺/CX3CR1⁺ M-MDSCs, as either ligand is sufficient to support their recruitment [98]. In a recent lung metastasis model, CCR1 deficiency markedly reduced the number of monocyte-derived cells within metastatic lungs, whereas CCR2 deficiency primarily impaired their initial mobilization. Combined blockade of CCR1 and CCR2 produced stronger inhibition of monocyte recruitment to lung metastases than targeting either axis alone [99].

Importantly, the systemic mobilization of bone marrow-derived myeloid cells not only shapes the primary tumor microenvironment but also contributes to the formation of the PMN. In this process, bone marrow-derived hematopoietic progenitors and myeloid populations, including neutrophils and monocytes, can be mobilized into the circulation and preferentially accumulate in distant tissues before overt metastatic lesions develop [7]. Early studies showed that VEGFR1-expressing bone marrow-derived hematopoietic progenitor cells accumulate at future metastatic sites prior to the arrival of disseminated tumor cells and preferentially localize to fibronectin-rich regions, thereby promoting the further recruitment of bone marrow-derived cells [6]. Tumor-derived systemic factors can further reinforce this process; for example, hypoxia-induced lysyl oxidase (LOX) released from primary tumors accumulates in distant organs, promotes collagen crosslinking and extracellular matrix remodeling, and facilitates the recruitment of CD11b^+^ bone marrow-derived myeloid cells [100]. These recruited cells can cooperate with local stromal and vascular components to further remodel the extracellular matrix, alter vascular permeability, and establish an immunosuppressive microenvironment [7, 101]. In parallel, mobilized HSPCs can populate pre-metastatic tissues and differentiate into immunosuppressive myeloid cells, facilitating subsequent metastasis [102]. Together, these systemic and local interactions prepare distant organs for the survival, seeding, and subsequent colonization of circulating tumor cells.

### Splenic extramedullary hematopoiesis and immune regulation

Beyond the bone marrow, tumor-driven hematopoietic remodeling can also extend to the spleen. The spleen is the largest secondary lymphoid organ, which normally filters the blood, removes senescent or damaged blood cells and circulating debris, and coordinates innate and adaptive immune responses against blood-borne antigens [103]. Under tumor-associated conditions, the spleen can additionally become an important site of extramedullary hematopoiesis. In a genetic mouse model of lung adenocarcinoma, HSPCs accumulate in the spleen and undergo myeloid-biased differentiation, providing an additional source of tumor-promoting monocytes and neutrophils [104]. Moreover, in mouse hepatoma models, the splenic microenvironment can educate recruited HSPCs toward tumor-promoting myeloid differentiation through CCL2/CCR2-dependent recruitment and local stromal signals [105]. Moreover, proliferating CCR2⁺ inflammatory monocytes accumulate in the splenic marginal zone, where they remodel the local architecture, interact closely with memory CD8⁺ T cells, and cross-present tumor antigens to induce T-cell tolerization [106]. Limited human tissue evidence is consistent with the occurrence of tumor-associated splenic extramedullary hematopoiesis: spleens resected from patients with several types of solid tumors contained increased numbers of CD133⁺ HSPC-like cells and CD11b⁺ myeloid cells, and a higher abundance of splenic CD133⁺ cells was associated with poorer survival in patients with gastric cancer [105]. Together, these findings identify the spleen not only as a complementary site of hematopoiesis beyond the bone marrow, but also as an important organ for tumor-driven myeloid remodeling and immune regulation, thereby contributing to systemic immune remodeling in cancer. However, these functions have not been directly established in humans, and species-specific differences in splenic hematopoiesis warrant caution when extrapolating findings from mice to humans.

### Therapeutic targeting of myelopoiesis and myeloid trafficking

Given the important role of tumor-driven myelopoiesis and aberrant myeloid trafficking in shaping the tumor microenvironment, multiple therapeutic strategies have been explored to interrupt these processes (Table 1). As discussed above, blockade of IL-1β and MMP-13 can partially restore tumor-altered granulopoiesis, highlighting the therapeutic potential of targeting upstream hematopoietic reprogramming. More broadly, the plasticity of hematopoietic progenitors may be therapeutically exploited through trained granulopoiesis. β-glucan-induced transcriptional and epigenetic reprogramming of granulopoietic progenitors can generate neutrophils with enhanced antitumor activity [107]. This concept has been further supported by bone marrow-targeted nanobiologics that induce trained myelopoiesis and enhance checkpoint inhibition [108], as well as by recent studies showing that Mycobacterium bovis Bacillus Calmette-Guérin (BCG), alone or combined with β-glucan, reprograms HSPCs and granulopoiesis to generate antitumor myeloid cells and improve tumor control [109, 110]. Importantly, cancer therapies themselves (chemotherapy, radiotherapy and immunotherapy) can also reshape bone marrow in distinct ways, highlighting the need to preserve or restore hematopoietic homeostasis during treatment. For example, chemotherapy-induced myelosuppression initiates compensatory hematopoietic regeneration, but the extent of bone marrow recovery depends on treatment intensity and duration. In addition to depleting hematopoietic cells, chemotherapy can disrupt the broader bone marrow microenvironment, affecting stromal and bone compartments and signaling pathways involved in HSC retention and regeneration, particularly the CXCL12-CXCR4 axis [111]. Radiotherapy can similarly damage the sinusoidal endothelial niche, and regeneration of this vascular compartment is required for efficient HSPC expansion and hematopoietic reconstitution [112]. In contrast, immune checkpoint inhibition (ICI) may partially reverse tumor-induced hematopoietic abnormalities; in mismatch-repair-deficient tumor models, short-term ICI partially restored bone marrow progenitor composition and the lymphoid-myeloid balance [113]. These findings also suggest opportunities to mitigate treatment-associated bone marrow dysfunction. Clinically, bone marrow-sparing radiotherapy has reduced hematological toxicity by limiting radiation exposure to hematopoietically active marrow [114]. Clearly, strategies aimed at preserving or restoring hematopoietic niche function warrant further investigation.

**Table 1:** Clinical trials targeting hematopoietic reprogramming and myeloid cell trafficking in solid tumors^1^

Strategy	Target	Drug name	Combination therapy	Tumor type	Phase	NCT number	Last reported status	Key reported outcome and safety
Hematopoietic reprogramming	IL-1β	Canakinumab	Pembrolizumab or canakinumab + pembrolizumab	Resectable stage IB–IIIA NSCLC	II	NCT03968419 (CANOPY-N)	Terminated	MPR was 2.9% with canakinumab, 17.1% with canakinumab + pembrolizumab, and 16.7% with pembrolizumab alone, with no unexpected safety signals [123]
	IL-1β	Canakinumab	Pembrolizumab + platinum-based chemotherapy	Previously untreated locally advanced/metastatic NSCLC	III	NCT03631199 (CANOPY-1)	Terminated	Canakinumab did not improve PFS or OS when added to pembrolizumab plus chemotherapy, with no unexpected safety signals [124]
Myeloid mobilization, trafficking, and maintenance	CXCR1/2	Navarixin	Pembrolizumab	NSCLC, castration-resistant prostate cancer, and MSS colorectal cancer	II	NCT03473925	Completed	Clinical activity was limited across tumor cohorts (ORR 0–5%), and toxicity was generally manageable [125]
	CXCR2	AZD5069	Enzalutamide	Metastatic castration-resistant prostate cancer	Ib/II	NCT03177187	Terminated	Composite responses occurred in 24% of evaluable patients; no DLTs occurred, although grade ≥ 3 neutropenia was frequent (48%) [116]
	CCR2/CCR5	BMS-813160	Nivolumab + gemcitabine + nab-paclitaxel	Borderline-resectable or locally advanced PDAC	I/II	NCT03496662	Completed	ORR was 42% in borderline-resectable and 20% in locally advanced PDAC, and treatment was well tolerated [126]
	CCR2/CCR5	BMS-813160	Chemotherapy or nivolumab, cohort-dependent	Advanced pancreatic and colorectal cancer	I/II	NCT03184870	Completed	In first-line PDAC, ORRs were 26–37% across BMS-813160-containing regimens versus 28% with chemotherapy alone; treatment was generally tolerable [127]
	CCR2	PF-04136309	FOLFIRINOX	Borderline-resectable or locally advanced PDAC	I	NCT01413022	Completed	Objective response and disease control rates were 49% and 97%, respectively, with no treatment-related deaths [128]
	CCR2	PF-04136309	Gemcitabine + nab-paclitaxel	Previously untreated metastatic PDAC	I/II	NCT02732938	Terminated	ORR was 23.8%, while pulmonary toxicity occurred in 24% of patients and no clear efficacy advantage was observed [129]
	CCL2	Carlumab (CNTO888)	Monotherapy	Metastatic castration-resistant prostate cancer	II	NCT00992186	Completed	No objective responses were observed; 34% achieved stable disease for ≥ 3 months, and treatment was generally well tolerated [120]
	CCL2	Carlumab (CNTO888)	Standard-of-care chemotherapy, cohort-dependent	Advanced solid tumors	I	NCT01204996	Completed	One partial response and stable disease in 38% of patients were reported; combination treatment was generally feasible [130]
	CCL2	Carlumab (CNTO888)	Monotherapy	Advanced solid tumors	I	NCT00537368	Completed	Carlumab was well tolerated, but no objective responses were observed [131]
	CCR5	Vicriviroc	Pembrolizumab	Advanced/metastatic MSS colorectal cancer	II	NCT03631407	Completed	ORR was 5% in both dose cohorts, with manageable toxicity [132]
	CCR5	Maraviroc	Pembrolizumab	Refractory MSS metastatic colorectal cancer	I	NCT03274804 (PICCASSO)	Completed	ORR was 5.3% and median OS was 9.83 months; treatment was feasible with limited severe toxicity [133]
	CSF1R	Pexidartinib	Neoadjuvant paclitaxel	Early breast cancer; I-SPY2 experimental arm	II	NCT01042379	Pexidartinib arm halted	Enrollment was halted after 9 patients because of severe hepatobiliary toxicity, preventing meaningful efficacy assessment [121]
	CSF1R	Pexidartinib (PLX3397)	Paclitaxel	Advanced solid tumors; ovarian-cancer expansion	I	NCT01525602	Completed	CR and PR occurred in 3% and 13% of evaluable patients, respectively; grade 3–4 AEs occurred in 70% [134]
	CSF1	Lacnotuzumab (MCS110)	Spartalizumab	Advanced melanoma, TNBC, pancreatic and endometrial cancers	I	NCT02807844	Completed	No objective responses were observed; one patient achieved prolonged stable disease, with no grade 4 or drug-related fatal AEs [135]
	CXCR4	Plerixafor (AMD3100)	Cemiplimab	Metastatic PDAC	II	NCT04177810	Completed	No objective responses were observed and DCR was 9.5%; treatment was generally well tolerated [122]
	CXCR4	Motixafortide (BL-8040)	Pembrolizumab ± chemotherapy	Metastatic PDAC	II	NCT02826486 (COMBAT)	Completed	With chemotherapy-containing treatment, ORR was 21.1% and DCR 63.2%; treatment was generally well tolerated [136]

^1^The table summarizes selected clinical trials evaluating therapeutic strategies targeting hematopoietic reprogramming or myeloid cell mobilization, trafficking, and maintenance in solid tumors. Reported clinical outcomes and major safety findings are presented based on published trial results. *AE*, adverse event; *CR*, complete response; *CRPC*, castration-resistant prostate cancer; *DCR*, disease control rate; *DLT*, dose-limiting toxicity; *MPR*, major pathological response; *MSS*, microsatellite stable; *MTD*, maximum tolerated dose; *NSCLC*, non-small cell lung cancer; *ORR*, objective response rate; *OS*, overall survival; *PDAC*, pancreatic ductal adenocarcinoma; *PFS*, progression-free survival; *pMMR*, proficient mismatch repair; *PR*, partial response; *RECIST*, Response Evaluation Criteria in Solid Tumors; *SD*, stable disease; *TNBC*, triple-negative breast cancer

In parallel, therapeutic strategies have also focused on blocking downstream myeloid trafficking. Among these, blockade of the CXCR2 axis, which mediates the recruitment of neutrophils and G-MDSCs, has shown relatively consistent efficacy. The oral CXCR1/2 allosteric inhibitor SX-682 enhances CD8⁺ T cell infiltration and suppresses tumor growth in melanoma models. In patients with metastatic melanoma refractory to anti-PD-1 and anti-CTLA-4 therapy, the combination of SX-682 with pembrolizumab achieved a 21% objective response rate (ORR) and a 63% disease control rate (DCR) (Clinical Trial:NCT03161431). Another small-molecule CXCR2 inhibitor, AZD5069, demonstrated reduced tumor burden and prolonged survival when combined with anti PD-1 therapy in a non-alcoholic steatohepatitis hepatocellular carcinoma model [115]. Besides, AZD5069 in metastatic castration-resistant prostate cancer in combination with androgen receptor inhibition (enzalutamide) has also shown good tolerability and antitumor activity [116].

Macrophages constitute the largest immune cell population within the tumor microenvironment, making them an attractive target for therapeutic intervention. However, it remains challenging, largely due to the extensive redundancy of their migratory and differentiation networks and the substantial risk of systemic toxicity. Preclinical studies showed that blocking GM-CSF/G-CSF could inhibit the accumulation of MDSCs, but has not yet translated into clinical outcomes [117]. Similarly, whereas CCR2 antagonists combined with anti-PD-1 therapy restored antitumor responses and improved efficacy in preclinical models [118, 119], their clinical translation has yielded more limited outcomes. In early pancreatic cancer trials, the CCR2 inhibitor PF-04136309, when combined with FOLFIRINOX chemotherapy, significantly reduced TAM infiltration, but failed to confer a clear survival benefit, despite acceptable overall tolerability. The anti-CCL2 monoclonal antibody carlumab (CNTO888) demonstrated acceptable safety but no clinical efficacy, accompanied by a rebound increase in circulating CCL2 levels in metastatic castration-resistant prostate cancer [120]. The CSF1/CSF1R axis, which regulates TAM survival and polarization, represents another major clinical focus. In the I-SPY2 trial for early breast cancer, enrollment in an experimental arm evaluating the CSF1R inhibitor pexidartinib in combination with neoadjuvant paclitaxel was halted due to a serious adverse event of vanishing bile duct syndrome [121]. In ICI-resistant solid tumors, the clinical trial of the CSF1R inhibitor cabiralizumab combined with nivolumab is still ongoing (Clinical Trial: NCT03336216). The CXCR4/CXCL12 axis, critical for myeloid cell homing, is also being explored therapeutically. The CXCR4 antagonist plerixafor (AMD3100) combined with the PD-1 inhibitor cemiplimab demonstrated safety but limited efficacy in a phase II pancreatic cancer trial, with only 2 (of 21) cases of disease stabilization [122]. Yet, CXCR4 antagonism led to increased mobilization of neutrophils, monocytes and lymphocytes into the blood and increased tumor inflammation. These monocytic populations also expressed alternative chemokine receptors including CCR2, CCR5, and CXCR2, suggesting compensatory migratory pathways [122].

Overall, although several strategies targeting myeloid recruitment and survival have entered clinical evaluation, evidence to date remains largely limited to early-phase trials, with clinical benefit varying across tumor types and treatment settings. Key translational challenges include redundancy and compensatory signaling among chemokine pathways, strong tumor-context dependency, and systemic toxicities associated with disruption of physiological myeloid functions.

## Peripheral blood as an interface for systemic tumor-host communication and disease monitoring

### Peripheral blood as a communication route

**Fig. 4:**
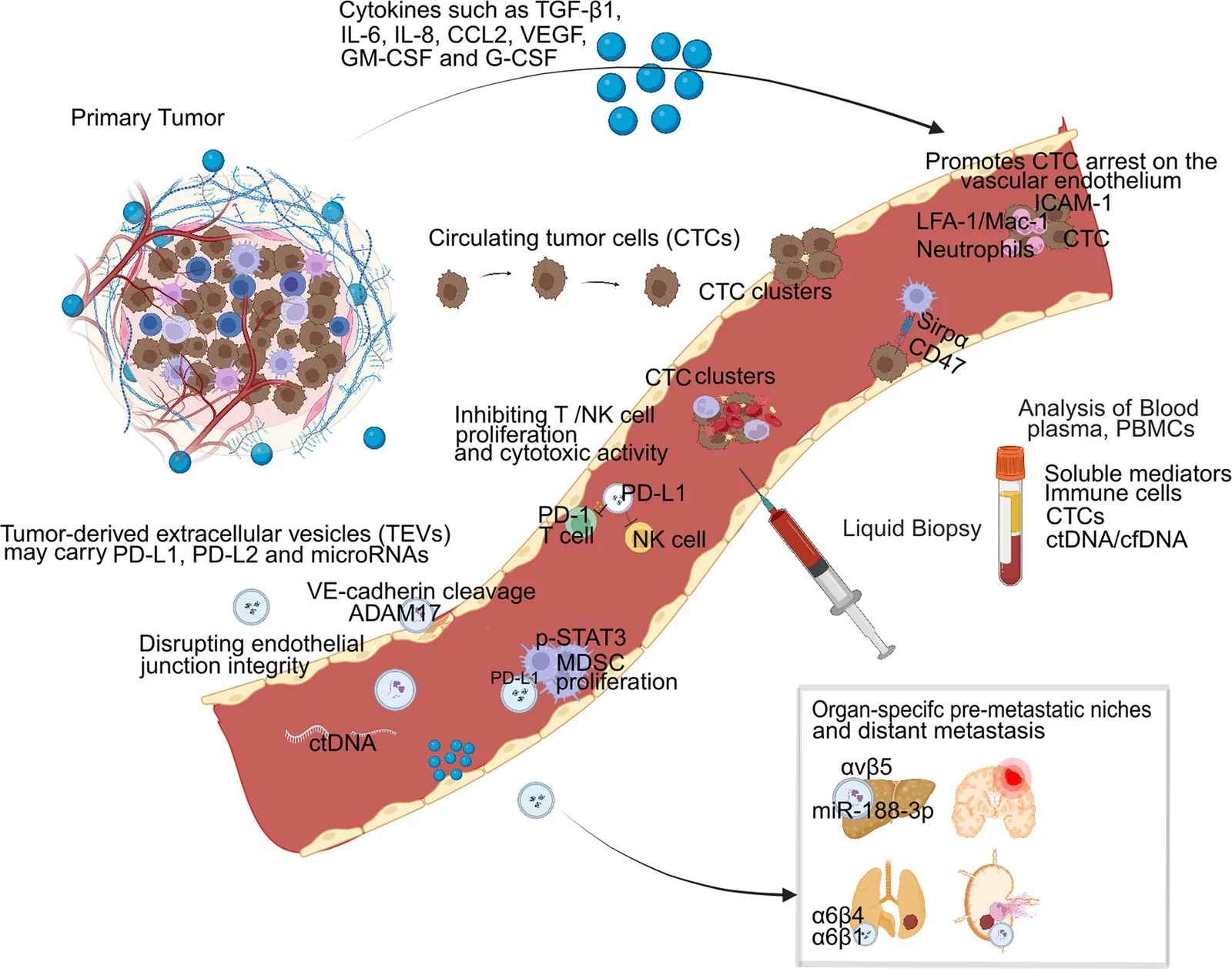
Tumor-derived circulating factors and cells orchestrate systemic immune modulation and metastatic dissemination. The figure illustrates how the primary tumor releases multiple circulating components, including TEV, circulating tumor DNA (ctDNA), and CTC, which altogether reprogram systemic immunity and promote metastasis. Created with BioRender.com

The peripheral circulatory system is a critical pathway for systemic tumor-host interactions. The various components within the bloodstream, including soluble mediators, tumor-derived extracellular vesicles (TEVs) and circulating tumor cells (CTCs) are now recognized as active participants in tumor-immune communication that contribute to the establishment of a pro-tumor systemic environment and facilitate metastatic progression (Fig. 4). Additionally, some of the circulating components (including tumor-metabolic products, soluble mediators, circulating suppressive immune cells, ctDNA/cfDNA, CTCs) also present a rationale for immune monitoring.

Considering that the recruitment of MDSCs and the establishment of  an immunosuppressive environment at loco-regional distal organs such as lymph nodes and bone marrow require the circulation of messengers, one would expect a measurable increase in certain cytokines and chemokines in the blood of cancer patients. Indeed, most reproducibly, elevated serum levels of the immune suppressive cytokine IL-6 were found to be prognostic, associated with progressive disease and reduced response to chemotherapy or immune checkpoint inhibitors [137–139]. Similarly, elevated IL-8 and VEGF were found in multiple studies, usually indicating poor prognosis [140–142]. However, although the experimental evidence for the CCL2/CCR2 axis in recruiting myeloid cells to the tumor is strong, studies measuring serum CCL2 yielded more heterogeneous results [142, 143]. Likewise, the evidence for GM-CSF and G-CSF elevation in serum seems context-dependent [144]. Conversely, circulating cellular signatures may also provide indirect information on immune remodeling occurring in distal compartments. In patients with solid tumors, circulating GMPs were increased by approximately four- to sevenfold and HSPCs displayed a marked granulocytic bias, suggesting that circulating progenitor composition may reflect tumor-associated alterations in bone marrow hematopoiesis [82]. Whether circulating biomarkers can similarly capture TDLN-specific immune activity remains less clear, although tumor-specific T-cell clonotypes shared between TDLNs and peripheral blood may provide a potential readout of LN-associated antitumor responses [35]. Interestingly, recent studies demonstrate that highly sensitive plasma proteomics can identify protein signatures associated with cancer risk years prior to diagnosis [40, 145]. Such protein signatures include immune-related proteins and may not only reflect changes in the primary tumor, but also derive from peripheral processes in LN and BM. Further research will be needed to establish whether such technology would also enable, for example, on-treatment monitoring of systemic immune function.

TEVs can carry a variety of bioactive molecules, such as nucleic acids, proteins, lipids and metabolites. These molecular cargoes determine how exosomes shape immune cell behavior in blood circulation and form PMNs. It was demonstrated that PD-L2 in exosomes derived from clear cell renal cell carcinoma and PD-L1 in exosomes secreted by prostate cancer cells can directly bind to PD-1 on the surface of T cells, significantly inhibiting T cell proliferation and cytotoxic activity [146, 147]. Gastric cancer-derived exosomes carried PD-L1, which mediated STAT3 signaling pathway in MDSCs, significantly promoting their proliferation [148]. In colorectal cancer, tumor-derived exosomes enriched in metalloproteinase ADAM17 can act on vascular endothelial cells, inducing VE-cadherin cleavage and thereby disrupting endothelial junction integrity [149]. This alteration markedly increases vascular permeability and provides a structural basis for the establishment of the pre-metastatic niche (PMN). Similarly, TEVs released by breast cancer cells carry microRNA miR-9-5p, which is preferentially taken up by Kupffer cells in the liver. By regulating cholesterol homeostasis-related pathways, they reprogram the metabolic state and inflammatory phenotype of Kupffer cells [150]. Similarly, miR-188-3p in colorectal cancer-derived exosomes can be taken up by hepatic stellate cells, inducing their activation and promoting extracellular matrix deposition. These all promote the formation of a liver-PMN [151]. Beyond soluble cargo, exosomes can also display integrins on their surface, which determine preferential uptake by specific cell types in distant organs. Hoshino *et al.* demonstrated that lung-tropic tumor exosomes are enriched in integrins α6β4 and α6β1 and are preferentially internalized by lung fibroblasts and epithelial cells. In contrast, liver-tropic exosomes predominantly express αvβ5 and are selectively taken up by Kupffer cells. Following uptake, exosomes activate Src phosphorylation and induce a pro-inflammatory S100 gene program, thereby establishing organ-specific pre-metastatic niches prior to tumor cell arrival [152].

### Peripheral blood as a window for longitudinal cancer monitoring

CTCs represent viable tumor cells released or shed into the bloodstream. CTCs face numerous challenges in the bloodstream, including immune clearance and mechanical shear stress, and the vast majority of CTCs are unable to successfully establish metastatic sites [153]. To survive under these hostile conditions, CTCs adapt to their environment by interacting with various immune cells in the circulatory system, such as neutrophils, macrophages, and NK cells, thereby avoiding immune elimination. Neutrophils can physically associate with CTCs through the interaction between LFA-1/Mac-1 receptors on neutrophils and ICAM-1 expressed on tumor cells. This interaction promotes CTC arrest on the vascular endothelium and enhances metastatic seeding efficiency [154]. Macrophages can directly interact with CTCs through SIRPα and CD47 to protect CTCs from phagocytosis [155]. They can also activate Notch signaling in CTCs, which enhances CTC survival and metastasis [156]. Furthermore, in colorectal cancer, it was shown that macrophages can induce the EMT program in the tumor to enhance CTC release into the bloodstream and thereby promote CTC-mediated metastasis [157]. Clinical studies have further established the translational relevance of CTCs. The CellSearch system has been extensively used to evaluate their prognostic significance. Early studies confirmed the presence of detectable CTCs in patients with urothelial carcinoma [158], while subsequent large-scale clinical analyses established CTC counts as an independent prognostic biomarker for multiple solid tumors [158–160]. Meta-analyses further demonstrated that CTC positivity is significantly associated with reduced overall survival and an increased risk of disease progression, highlighting CTCs’ critical role as surrogate markers of tumor invasiveness and systemic metastasis [160, 161]. In large breast cancer cohorts, stratification by CTC count thresholds (≥ 1, ≥ 2, ≥ 5 per 7.5 mL of blood) revealed a stepwise decrease in overall survival (OS) [162].

From a clinical perspective, longitudinal immune monitoring based on peripheral blood enables real-time assessment of tumor progression-related immune modulation and the evolution of the systemic immune status during treatment. Supporting this concept, Van den Ende et al. showed in the PERFECT cohort of resectable esophageal adenocarcinoma patients treated with neoadjuvant PD-L1 blockade that non-pathological complete responders displayed a systemic immunosuppressive profile at baseline, including increased Tregs and intermediate monocytes, reduced type-2 conventional dendritic cells, elevated immunosuppressive cytokines, and further expansion of activated Tregs during treatment, which was associated with EMT in the tumor [163]. In another study of prostate cancer patients treated with GVAX and ipilimumab, it was demonstrated that peripheral dendritic cell activation and MDSC frequencies were associated with clinical outcome, further supporting the value of blood-based myeloid profiling in immunotherapy [164]. Consistently, a comprehensive peripheral blood immunoprofiling study across multiple cancer types identified distinct outcome-associated immunotypes: patients characterized by activated CD8⁺ T cell dominance displayed higher response rates and longer survival, whereas those dominated by myeloid-derived immunosuppression (high MDSC frequencies and low lymphocyte ratios) showed the poorest outcomes [165]. Additional studies further support this concept, showing that persistently elevated peripheral M-MDSCs in metastatic melanoma and sustained lymphopenia during immune checkpoint blockade were associated with worse therapeutic outcomes [166, 167].

Measuring ctDNA can be a valuable noninvasive tool in postoperative adjuvant treatment decision-making, prognostic assessment, early relapse detection, resistance monitoring, and evaluation of therapeutic efficacy. ctDNA carries information on genomic or epigenetic abnormalities, such as mutations in specific driver genes (e.g., EGFR, ROS1, *KRAS*, *NRAS*, *BRAF*, or *TP53*), chromosomal rearrangements, copy number changes, and methylation markers [168–170]. Taking colorectal cancer as an example, the DYNAMIC phase III clinical trial demonstrated that a ctDNA-guided adjuvant therapy strategy (based on the detection of tumor-specific somatic mutations in plasma) could significantly reduce the use of chemotherapy without compromising relapse-free survival (RFS) [171]. Consistently, the GALAXY study reported that ctDNA positivity at 4 weeks after surgery was strongly associated with an increased risk of recurrence and represented the most powerful prognostic factor for relapse among stage II and III colorectal cancer patients [172]. Furthermore, a meta-analysis including 65 cohort studies confirmed that postoperative ctDNA positivity was significantly associated with shorter RFS and overall survival (OS) [173]. ctDNA conversion to positivity often preceded radiological recurrence by several months, underscoring its potential in early relapse detection [173]. In non-small cell lung cancer (NSCLC), ctDNA dynamics during pembrolizumab treatment showed a high concordance with radiographic response and provided earlier indication of treatment efficacy and disease progression [174]. Moreover, evidence from large longitudinal studies such as TRACERx further supports the application of ctDNA for monitoring tumor clonal evolution and minimal residual disease (MRD), offering new opportunities for personalized treatment optimization and early therapeutic intervention [175]. Several liquid biopsy platforms measuring ctDNA as a biomarker, such as the cobas® EGFR Mutation Test v2, Guardant360® CDx, and FoundationOne®, have been FDA-approved for clinical cancer management, showing great promise in early diagnosis, minimal residual disease (MRD) detection, therapeutic monitoring, and prognostic assessment [175–177]. Their sensitivity, specificity, and standardization of data interpretation remain insufficient to support widespread clinical implementation.

## Conclusion

In summary, although lymph nodes, bone marrow, and the circulation are discussed separately for clarity, these compartments are functionally interconnected within the broader systemic response to tumor development. Tumor-derived signals can remodel TDLNs and bone marrow hematopoiesis, alter the composition and functional state of circulating immune cells, and facilitate tumor growth, PMN formation, and subsequent metastatic colonization. In turn, immune cells originating from TDLNs and the bone marrow can enter the circulation and subsequently influence tumor progression, metastasis, and responses to anticancer therapies. Together, these interactions form a dynamic systemic network that evolves throughout tumor development. Such dynamic immune remodeling also poses common therapeutic and monitoring challenges, including pathway redundancy and compensation, tumor- and patient-specific heterogeneity, limited numbers of currently known compartment-specific biomarkers, and difficulties in longitudinally assessing immune changes across different tissues. A more integrated understanding of these interconnected compartments may therefore improve our understanding of systemic tumor-immune interactions and support the development of more precise therapeutic and biomarker strategies.

## Data Availability

No datasets were generated or analysed during the current study.
